# Road traffic fatalities - a neglected epidemic in rural northern Ghana: evidence from the Navrongo demographic surveillance system

**DOI:** 10.1186/s40621-014-0022-3

**Published:** 2014-09-21

**Authors:** Ayaga Bawah, Paul Welaga, Daniel K Azongo, George Wak, James F Phillips, Abraham Oduro

**Affiliations:** 1 Mailman School of Public Health, Columbia University, New York, NY 10032 USA; 2 Navrongo Health Research Centre, P. O. Box 114, Navrongo, Ghana

**Keywords:** Road, Traffic, Fatalities, Neglected epidemic, Verbal autopsy, Demographic surveillance systems

## Abstract

**Background:**

Globally, road traffic fatalities have been on the increase, particularly in low-and-middle income countries. Much of this is attributed to increases in the acquisition, and use of motorized vehicles. However, there is very little empirical research to understand the causes and determinants of this threat. This paper investigates time trends and determinants of road traffic accidents in the Kassena-Nankana district of northern Ghana.

**Methods:**

First, we utilized causes of death data gathered by the Health and Demographic Surveillance System in Navrongo, to examine trends in deaths due to injury, particularly those related to road traffic crashes. Subsequently, we employed multivariate logistic regression to examine factors associated with deaths due to all injuries and road traffic crashes among adults 15–59 years of age.

**Results:**

Results show a three-fold increase in mortality (from less than 6% in 1995 to about 18% in 2010) due to injuries in the Kassena-Nankana district in about a decade. Fatalities resulting from road traffic crashes constitute the greatest share of the burden of mortality resulting from injuries. Increases in road traffic fatalities have coincided with recent increases in motor and vehicular traffic in the region. Several factors are associated with the increased risk of deaths from road traffic accidents, principal among which include urban residence (OR = 1.74 95% CI 1.09-2.78), being male and in the prime adult ages of between 20–29 years old (OR = 4.85 95% CI 2.65-8.89), as well as people with higher levels of education (OR = 3.21 95% CI 1.75-5.87) and those in higher socioeconomic status categories (OR = 2.43 95% CI 1.21-4.88).

**Conclusions:**

Results suggest that road traffic fatalities have become a major cause of morbidity and mortality and brings into focus the need for measures to curb this looming crisis. There is need for strategic interventions to be adopted to avert what is sure to become one of the leading causes of death in this impoverished locality.

## Background

Worldwide, road traffic injuries (RTI) have increased contributing to a significant proportion of the burden of morbidity and mortality. For instance, in 2006 the Population Reference Bureau (PRB) reported that an estimated 1.2 million people are killed annually in road traffic crashes (Worley 2006). The World Health Organization (WHO) in its 2009 global status report on road safety reaffirmed this when it noted “Approximately 1.3 million people die each year on the world's roads, and between 20 and 50 million sustain non-fatal injuries” (World Health Organization 2009).

Incidentally, developing countries bear the largest share of the burden (Nantulya and Reich 2002). More recently, the WHO has predicted that road traffic accidents will rise to become the fifth leading cause of death in 2030, from a ninth position in 2004 (World Health Organization 2011).

Sadly, the sub-group of the population that is most affected is, the young and most active adult members of the population, often described as the most productive population. Literature reviewed across different countries and settings consistently show that people in the age groups 15–49 or 15–59 are more prone to road traffic fatalities (Bachani et al. 2012; Ditsuwan et al. 2011; Herman et al. 2012; Garrib et al. 2011). Males however, disproportionately bear a higher brunt of the share of road traffic accidents and fatalities than females (Garrib et al. 2011; Ditsuwan et al. 2011).

Apart from the costs associated with road traffic accidents in terms of the toll in human lives, the economic burden it puts on economies is high. For instance, a 2004 World report on road traffic injuries and fatalities summarized the economic cost in the following text that the *“…economic cost of road crashes and injuries is estimated to be 1% of gross national product (GNP) in low-income countries, 1.5% in middle-income countries and 2% in high-income countries. The global cost is estimated to be US$ 518 billion per year. Low-income and middle-income countries account for US$ 65 billion, more than they receive in development assistance”* (Peden et al. 2004). In Ghana, statistics from the National Road Safety Commission show that the country loses over $165million of the Country’s Gross Domestic Product (GDP) to road accidents (Kudebong et al. 2011).

Despite the emerging evidence, there is lack of empirical data to actually characterize the burden and risk factors associated with road traffic fatalities, especially in low- and middle-income countries (Ameratunga et al. 2006; Peden and Toroyan 2005). The aim of this paper is to use health and demographic surveillance data to describe the burden of road traffic accidents for the period 1995–2011 and examine the risk factors associated with it.

### Data and methods

#### Setting

This study focuses on the Kassena-Nankana district of northern Ghana where social and economic conditions are among the worst in the country (Insert is Map of Ghana showing location of KND). Prior to health interventions introduced in the district by the Navrongo Health Research Center, both childhood and adult mortality were among the highest in the country. While social and economic conditions have remained generally poor, data analyzed from the demographic and health surveillance show that mortality has declined considerably in the district over the past decade (Oduro et al. 2012; Azongo et al. 2012) Figure 1.

**Figure 1:**
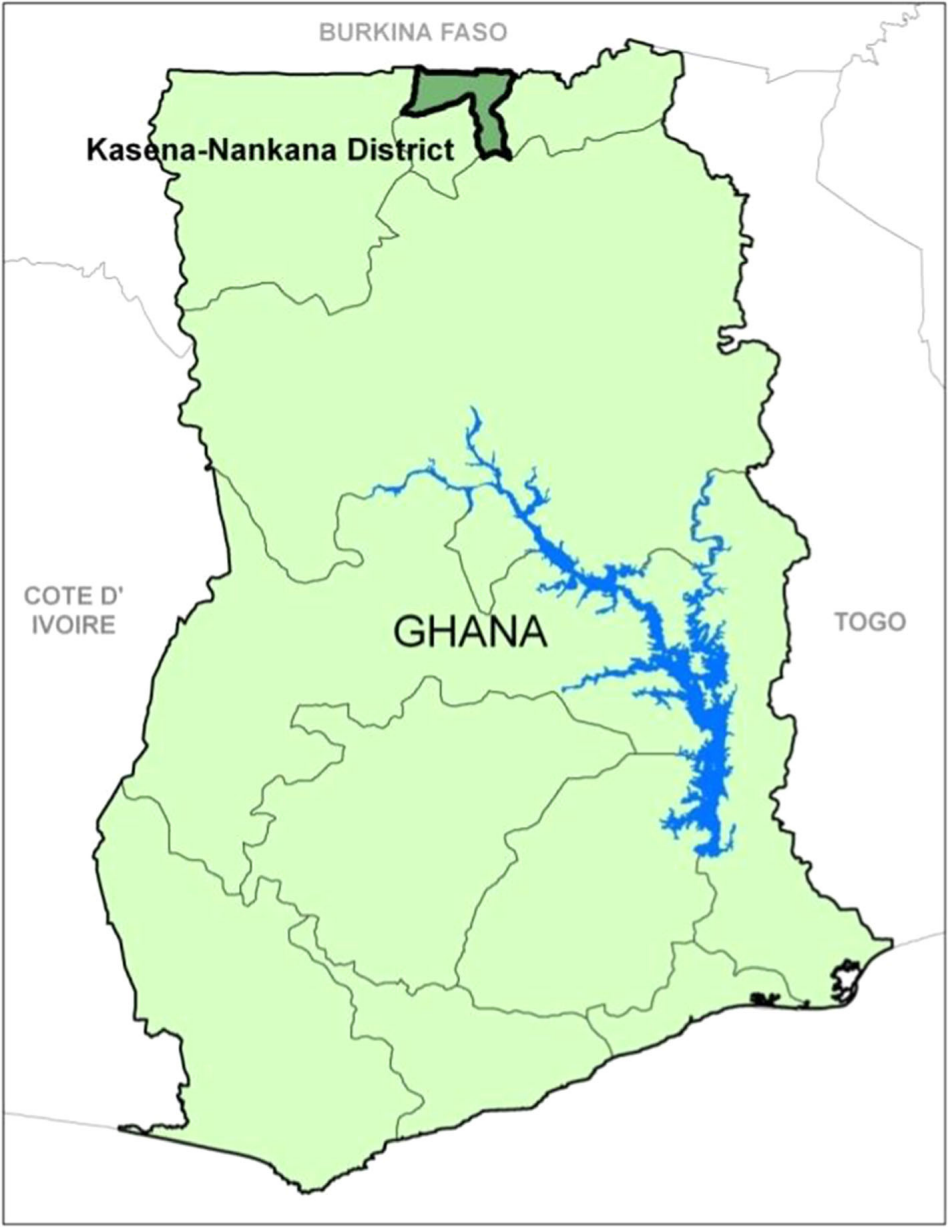
**Map showing location of Kassena-Nankana district.**

Similarly, fertility levels have declined over the same period (Phillips et al. 2012; Debpuur et al. 2002; Oduro et al. 2012). For instance, while infant mortality decreased from 129 deaths per 1,000 live births in 1995 to 85 in 2003 representing a 34 percent decrease, under-five mortality declined from about 147 to 83 per 1000 live births, representing a 44 percent decline over the period (Binka et al. 2007). However, while mortality in general is declining recent data show as a share of the causes of death injury related deaths particularly those associated with motor traffic accidents have been increasing.

#### Data

We used causes of death data from the Navrongo Health and Demographic Surveillance system, a system of continuous assessment of demographic dynamics in Kassena-Nankana District (Binka et al. 1999). In 1993, a census of the entire population of the district was conducted and subsequently thereafter, at quarterly intervals field workers visit all dwelling units in the district to update information on demographic events such as births, deaths, and in- and out-migrations, including changes in household dynamics.

When a death is reported, relatives of the dead are visited by trained field workers to conduct an oral postmortem interview known as verbal autopsy (VA) to ascertain information on the circumstances leading to the death using a standardized questionnaire (Baiden et al. 2007). A panel of three or more physicians are constituted to independently review such interviews to determine the probable cause of death (Setel et al. 2005; Baiden et al. 2007; Bawah and Binka 2007). A death is assigned a specified cause based on the congruence of at least two or more physicians. The cause of death data presented in this paper cover the period 1995–2010.

We supplemented the HDSS data with publicly available data on road traffic accidents from the Motor-Transport and Traffic Unit (MTTU) of the Ghana Police Service and Driver and Vehicle Licensing Authority (DVLA). We use the MTTU and DVLA data in order to provide context for the analysis.

#### Data limitations

Given the general paucity of data on causes of death in developing countries, we tried as to utilize as much data as is available to us for the analysis. To this end, data for different periods are used for different aspects of the analysis. For instance, in conducting the regression analysis, we restricted the analysis to the period 1998–2011. The reason is that, prior to 1998 the DSS data did not collect information on many of the variables that we have included as covariates in the model. Also, prior 2006 Road Traffic Accidents as a separate cause of death did not exist. It was always lumped together with all other injuries and coded broadly as Injury deaths. However, from 2006 road traffic deaths were isolated and coded as a separate category which also explains why we restricted the analysis of the risk factors for road traffic accidents to the period 2006–2011. Similarly, the data on vehicle and motorcycle registration covered different periods because that was what was available at the DVLA and MTTU.

## Methods

First, we examined the distribution of injury deaths by specific cause and then show trends in injury mortality over the period 1995–2010, for all ages and then for adults 15–59 years old. Subsequently, we employed multivariate logistic regression to examine factors associated with deaths due to all injuries and specifically for those related to road traffic accidents, among adults 15–59 years of age. We employed logistic regression analysis because we created dichotomous dependent variables where we first looked at all injury deaths compared with all other causes combined and then road traffic deaths verses all other causes combined. Applying maximum likelihood we estimate parameters of the logistic regression model given by:

$$ logit\left({P}_i\right)= \ln \left(\frac{P_i}{P_i-1}\right) = \alpha + {\displaystyle {\sum}_{j=1}^3{\beta}_{1j}\left(e{d}_i\right)+{\beta}_2(male)}\kern0.5em +{\displaystyle {\sum}_{k=1}^5{\beta}_{3k}{(SES)}_k + {\beta}_4 rural} + {\displaystyle {\sum}_{l=1}^2{\beta}_{5l}{(period)}_l + {\beta}_6\left( dry\  season\right)}\kern0.5em +\kern0.5em {\displaystyle {\sum}_{m=1}^4{\beta}_{7m}{(age)}_m} + {\displaystyle {\sum}_{n=1}^{11}{\beta}_{8n}{(month)}_n} $$

Where *P*_*i*_ represents the dependent variable which takes the value of 1 if the event occurs, otherwise 0 and each β represents the effect of the corresponding control variable among eight specified background characteristics included in the model: i) maternal educational attainment, ii) sex, iii) socio-economic status, iv) rural versus urban residence, v) time period for the study, vi) season, vii) age group, and viii) month of observation. As noted earlier, we used data for the period 1998–2011 for all the injury deaths combined and for the road traffic accident deaths we used data for the period 2006–2011. In analysing the risks factors we restricted our analysis to only adults 15–59 years of age because majority of injury deaths that are attributed to road traffic fatalities are among adults (45.5%). We then modelled the impact of various background and socio-economic status factors on the dependent variable using simple logistic regression. Regression analysis were restricted to the period 1998–2011

## Results

**Figure 2:**
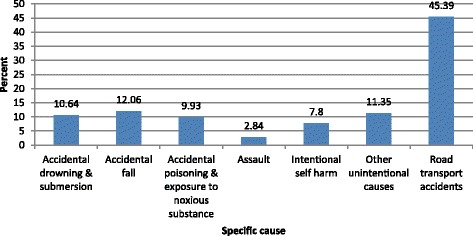
**Distribution of injury deaths by specific causes among adults aged 15–59 years in Navrongo HDSS: 2006–2011.**

From 2006 when road traffic deaths were isolated from all other injuries, there a total of 6643 deaths over that period out of which 282 (10.7%) were attributable to all injuries. However, of the injury deaths, 128 (45.5) of them were due to road traffic fatalities alone (see Figure 2). Clearly, road traffic crashes constitute the greatest share of the burden of injury mortality in this area and it is for this reason that we focused this paper on road traffic crashes as a share of overall injury mortality.

**Figure 3:**
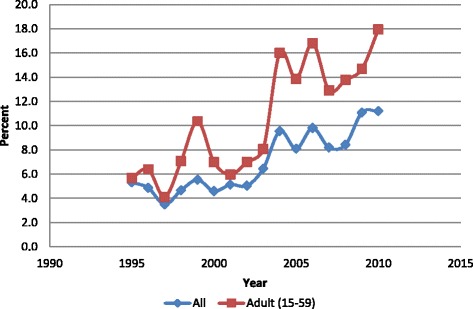
**Trends in injury mortality in the Kassena-Nankana District 1995–2010.**

Figure 3 shows time trends in injury mortality for the period 1995–2010. Over the period, there has been a significant increase in injury deaths, from a mere 6 per cent of all deaths in 1995 to about 18 per cent in 2010, representing a three-fold increase in about a decade. If we breakdown the figures separately into children (0–14) and adults (15–59), as would be expected, the prevalence is much higher among the adults population compared to persons below fifteen years of age. This is not surprising since by law children below 18 years of age are not allowed to drive in Ghana. Nonetheless, there is also a significant increase in childhood mortality resulting from injuries, as seen Figure 3.

**Figure 4:**
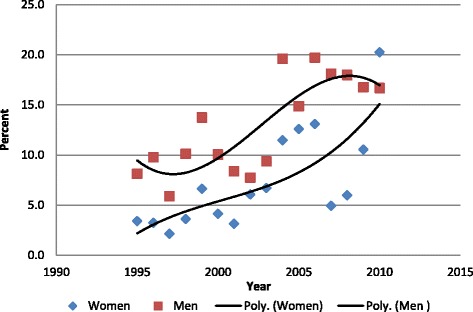
**Trends in injury deaths among adults 15–59 years old by sex in the Kassena-Nankana District 1995–2010.**

With respect to gender, the proportion is higher among males than females but over time there seem to be convergence, as shown by the third degree polynomial curves below (Figure 4). This suggests that deaths from road traffic crashes among females are gradually levelling off with that of the males. This is not surprising, given that many more females are beginning to ride motorbikes than has been in the past, exposing them to equally high risks of road traffic crashes and mortality.

Table 1 presents background characteristics and logistic regression results of adults 15–59 over the 1998–2011 for all deaths due to Injury in the Kassena-Nankana district. Overall, a total of 7465 adult deaths (15–59) were recorded, of which 705(9.44%) were due to injuries and road traffic crashes. The most significant predictors of injury and accident mortality include age of the individual, education, place of residence (rural/urban), socioeconomic status index (SES), time period, and month of year. For instance, the odds of dying from injury and road traffic crashes are significantly higher among adolescents (15–19) and young adults (20–24) in their most productive ages compared to older adults (50–59 year olds).

**Table 1:** **Background characteristics of participants, Unadjusted and Adjusted odds ratios of Injury deaths among adults aged 15 to 59 years in Navrongo HDSS: 1998-2011**

**Variable (col 1)**	**Deaths n (%) (Col 2)**	**Injury deaths n(%) (Col 3)**	**Unadjusted odds ratio of Injury deaths (95% CI) (Col 4)**	**Adjusted odds ratio (95% CI) (Col 5)**
**Education level**			1	
No education	3904 (52.3)	270 (6.9)		1
Primary/JSS	2282 (30.6)	283 (12.4)	1.91 (1.60 – 2.270***	1.0 (0.82 - 1.22)
Sec/Tertiary	643 (8.6)	117 (18.2)	2.99 (2.37 – 3.79)***	1.42 (1.08 - 1.87)*
Missing	636 (8.5)	35 (5.5)	0.78 (0.54 – 1.13)	0.52 (0.36 - 0.75)
**Sex**			1	
Female	3229 (43.3)	211 (6.5)		1
Male	4236 (56.7)	494 (11.7)	1.89 (1.60 – 2.23)***	1.85 (1.55 - 2.21)***
**Wealth index**				
Poor	1624 (21.8)	115 (7.1)	1	1
Next poor	1562 (20.9)	138 (8.5)	1.22 (0.94 - 1.58)	1.24 (0.95 - 1.62)
Average	1566 (21.0)	151 (9.6)	1.40 (1.09 - 1.80)**	1.41 (1.08 - 1.83)*
Less poor	1533 (20.5)	150 (9.8)	1.42 (1.11 - 1.84)**	1.31 (1.00 - 1.71)
Least poor	1066 (14.3)	149 (14.0)	2.13 (1.65 - 2.75)***	1.55 (1.14 - 2.12)**
Missing	114 (1.5)	7 (6.1)	0.86 (0.39 - 1.89)	1.05 (0.47 – 2.37)
**Place of residence**				
Rural	6370 (85.3)	546 (8.6)	1	1
Urban	1095 (14.7)	159 (14.5)	1.81 (1.50 – 2.19)***	1.39 (1.10 – 1.76)**
**Period of study**				
1998-2003	3973 (53.2)	298 (7.5)	1	1
2004-2007	1576 (21.1)	215 (13.6)	1.95 (1.62 – 2.35)***	1.87 (1.54 - 2.27)***
2008-2011	1916 (25.7)	192 (10.0)	1.37 (1.14 – 1.66)**	1.26 (1.03 - 1.54)*
**Season**				
Rainy	3550 (47.6)	338 (9.5)	1.02 (0.87-1.19)	NS
Dry	3915 (52.4)	367 (9.4)	1	
Age Group				
15-19	404 (5.4)	81 (20.1)	4.67 (3.48 - 6.27)***	5.11 (3.71 - 7.04)***
20-29	897 (12.0)	161 (18.0)	4.07 (3.21 - 5.17)***	4.22 (3.27 - 5.44)***
30-39	1421 (19.0)	172 (12.1)	2.56 (2.04 - 3.23)***	2.49 (1.96 - 3.16)***
40-49	1898 (25.4)	146 (7.7)	1.55 (1.22 - 1.97)***	1.48 (1.16 - 1.89)**
50-59	2845 (38.1)	145 (5.1)	1	1
**Month**				
January	701 (9.4)	45 (6.4)	1	1
February	619 (8.3)	51 (8.2)	1.31 (0.86 - 1.98)	1.24 (0.81 - 1.90)
March	691 (9.3)	56 (8.1)	1.29 (0.86 - 1.93)	1.19 (0.78 - 1.80)
April	641 (8.6)	83 (13.0)	2.17 (1.48 - 3.17)***	2.07 (1.40 - 3.06)***
May	579 (7.8)	63 (10.9)	1.78 (1.19 - 2.65)**	1.61 (1.07 - 2.44)*
June	555 (7.4)	42 (7.6)	1.19 (0.77 - 1.85)	1.10 (0.70 - 1.71)
July	522 (7.0)	56 (10.7)	1.75 (1.16 - 2.64)**	1.57 (1.03 - 2.40)*
August	612 (8.2)	66 (10.8)	1.76 (1.19 - 2.62)**	1.61 (1.08 - 2.42)*
September	622 (8.3)	50 (8.0)	1.27 (0.84 - 1.94)	1.14 (0.75 - 1.76)
October	660 (8.8)	61 (9.2)	1.48 (0.99 - 2.22)	1.50 (1.00 - 2.26)
November	558 (7.5)	53 (9.5)	1.53 (1.01 - 2.31)*	1.36 (0.89 - 2.08)
December	705 (9.4)	79 (11.2)	1.84 (1.26 - 2.70)**	1.76 (1.19 - 2.61)**
**Total**	7465 (100)	705 (9.44)		

Note: *P < 0.05 **P < 0.01 ***P < 0.001 NS - not significant at bivariate analysis.

Similarly, individuals with secondary or tertiary education relative to those without education, urban residents and those with progressively higher SES, all tend to have significantly higher odds of dying from injury related causes. In addition, time period is a significant predictor of injury and road traffic crashes. For instance, relative to the 1998–2003 period the odds of dying from road traffic accidents significantly increases statistically with time (odd ratio of 1.87 for the 2004–2007 and an odds ratio of 1.26 for the 2008–2011 period). What this means is that over time injury and road traffic fatalities have been on the rise. However, although males have higher odds of mortality from injuries and road traffic crashes the differences are not statistically significant.

These differences persist even after controlling for the confounding effects of all other covariates (Column 5, Table 1).

Now, since our primary focus in this paper is mortality resulting from road traffic crashes we isolated deaths due to road traffic crashes as a separate cause of death and modelled the effects of individual level and household level on risk of dying from road traffic crashes. Results for this analysis are presented in Table 2.

**Table 2:** **Risk factors for road traffic accidents (RTAs) among adults aged 15 to 59 years in Navrongo HDSS: 2006-2011**

**Variable**	**Deaths n (%)**	**Road traffic deaths n (%)**	**Odds ratio (95% CI)**	**Odds ratio (Adjusted (95% CI)**
**Education level**				
No education	1160 (43.9)	21 (1.8)	1	1
Primary/JSS	1026 (38.8	49 (4.8)	2.72 (1.62 – 4.57)***	1.44 (0.82 – 2.51)
Sec/Tertiary	337 (12.8)	49 (14.5)	9.23 (5.44 – 15.63)***	3.21 (1.75 – 5.87)***
Missing	120 (4.5)	9 (7.5)	4.40 (1.97 – 9.83)***	2.11 (0.90 – 4.95)
**Sex**				
Female	977 (37.0)	29 (3.00	1	1
Male	1666 (63.0)	99 (5.9)	2.07 (1.35 – 3.15)**	1.90 (1.21 – 2.98)**
**Wealth index**				
Poor	562 (21.3)	16 (2.9)	1	1
Next poor	547 (20.7)	14 (2.6)	0.90 (0.43 – 1.85)	0.93 (0.44 – 1.95)
Average	563 (21.3)	15 (2.7)	0.93 (0.46 – 1.91)	0.88 (0.42 – 1.83)
Less poor	592 (22.4)	34 (5.8)	2.08 (1.13 – 3.81)*	1.63 (0.86 – 3.12)
Least poor	365 (13.8)	48 (13.2)	5.17 (2.89 – 9.25)***	2.43 (1.21 – 4.88)*
Missing	14 (0.5)	1 (7.1)	2.63 (0.32 – 21.3)	1.20 (0.13 – 10.99)
**Place of residence**				
Rural	2233 (84.5)	79 (3.5)	1	1
Urban	410 (15.5)	49 (12.0)	3.70 (2.55 – 5.38)***	1.74 (1.09 – 2.78)*
**Period of study**				
2006-2008	1232 (46.6)	53 (4.3)	1	NS
2009-2011	1411 (53.4)	75 (5.3)	1.25 (0.87 – 1.79)	
**Season**				
Rainy	1310 (49.6)	55 (4.2)	1	NS
Dry	1333 (50.4)	73 (5.5)	1.32 (0.93 – 1.90)	
Age Group				
15-19	112 (4.2)	6 (5.4)	2.78 (1.09 – 7.07)*	3.02 (1.13 – 8.13)*
20-29	307 (11.6)	35 (11.4)	6.32 (3.59 – 11.12)***	4.85 (2.65 – 8.89)***
30-39	503 (19.0)	42 (8.4)	4.46 (2.59 – 7.69)***	3.45 (1.95 – 6.11)***
40-49	719 (27.2)	25 (3.5)	1.77 (0.98 – 3.21)	1.54 (0.83 – 2.85)
50-59	1002 (37.9)	20 (2.0)	1	1
**Month**				
January	215 (8.1)	4 (1.8)	1	1
February	214 (8.1)	14 (6.5)	3.69 (1.20 – 11.41)*	3.56 (1.12 - 11.31)*
March	235 (8.9)	11 (4.7)	2.59 (0.81 – 8.26)	2.81 (0.86 - 9.25)
April	237 (9.0)	18 (7.6)	4.34 (1.44 – 13.02)**	4.73 (1.53 - 14.64)**
May	210 (8.0)	17 (8.1)	4.65 (1.54 – 14.05)**	3.84 (1.23 - 12.01)*
June	230 (8.7)	7 (3.1)	1.66 (0.48 – 5.74)	1.44 (0.40 - 5.12)
July	195 (7.4)	6 (3.1)	1.67 (0.47 – 6.02)	1.52 (0.41 - 5.67)
August	220 (8.3)	9 (4.1)	2.25 (0.68 – 7.42)	2.00 (0.59 - 6.81)
September	223 (8.4)	9 (4.0)	2.22 (0.69 – 7.31)	1.97 (0.58 - 6.71)
October	233 (8.8)	7 (3.0)	1.63 (0.47 – 5.66)	1.60 (0.45 5.73)
November	189 (7.1)	10 (5.3)	2.95 (0.91 – 9.56)	2.98 (0.89 - 9.97)
December	242 (9.2)	16 (6.6)	3.73 (1.23 – 11.35)*	3.66 (1.16 - 11.53)*
**Total**	2643 (100)	128 (4.8)		

Note: *P < 0.05 **P < 0.01 ***P < 0.001 NS - not significant at bivariate analysis.

As in the case of all injury deaths, age, sex and level of education are significant predictors of road traffic fatalities. As would be expected, age is an important risk factor for road traffic crashes. Compared to individuals who are above age 50, those in the young adult ages have significantly higher risks of mortality from road traffic crashes, with those in the age groups 20–29 having the highest odds of dying from road traffic accidents. These differences persist and remain significant after controlling for the confounding effects of other covariates. Males also have significantly higher odds of mortality from road traffic crashes compared to females. Males, for instance, are 1.90 times more likely to die from road traffic crashes compared to their female counterparts controlling for the effects of other covariates. Also, individuals with secondary or higher education have significantly higher odds of dying from road traffic crashes compared to those without education, even after controlling for the effects of other covariates.

Other factors that significantly influence mortality from road traffic crashes include place of residence, socioeconomic status and month of the year. For instance, the odds of dying from road traffic crashes are significantly higher during the month of December compared to January, controlling for the effects of other variables. Similarly, the odds of dying from road traffic crashes is significantly higher in March/April compared to January and this seem to coincide with the Easter festivities. Also, the least poor tend to have significantly higher odds of mortality from road traffic crashes compared to the poorest group, even after controlling for all other variables.

## Discussion and conclusion

This paper examined trends and determinants of road traffic fatalities in the Kassena-Nankana district of rural northern Ghana where emerging evidence suggests rising levels of mortality from road traffic crashes. Using verbal autopsy data on causes of death we modelled the effects of various factors on all-injury and deaths resulting from road traffic crashes separately.

Results show a three-fold increase in mortality due to injury within a decade and road traffic fatalities constitute the greatest share of this burden. Increases in road traffic fatalities have coincided with recent increases in motor and vehicular traffic in the region. This observation is consistent with studies conducted elsewhere that have shown that increasing motorization in many parts of the developing world have largely been associated with increased rates of road traffic crashes (Al-Reesi et al. 2013; Han 2010; Islam and Al Hadhrami 2012). Thus with increasing influx of cheap motorbikes from China and India in the last few years it is not surprising that road traffic fatalities have been on the increase. The results also show that males tend to have higher risk of dying from road traffic crashes which is not surprising because until recently females rarely drove or rode motorbikes in this part of Ghana. However, in recent times many females are increasingly driving or riding motorcycles which now predisposes them also to accidents from these motorcycles and vehicles. As can be observed from the Figure 4, with passage of time there seem to be convergence where female mortality from road traffic accidents is gradually closing up with males.

Our data also show that the burden of road traffic mortality is highest among adults aged 20–39 years and young people aged 15–19 years, compared to adult 50–59 years of age. This is consistent with a study conducted in Kenya (Odero et al. 2003), which show that more than 75% of road traffic casualties are among the economically “productive” young adults.

As expected, individuals residing in urban areas have significantly higher risk of mortality compared to those in rural districts (Odero et al. 2003). This is not surprising because the increasing motorization which is partly attributable to the rising trend in road traffic fatalities is concentrated in the urban areas of the district, mainly in the Navrongo municipality and Paga, an urban town situated at the border between Ghana and Burkina Faso. Similarly, individuals with secondary or higher education and those in the highest socioeconomic status category are most at risk of dying from road traffic crashes. Again, this result is to be expected since people who are economically sound are those who are able to acquire the motorcycles and vehicles and thus more at risk of dying from road traffic crashes. Those with higher levels of education are also more likely to be from high SES households.

It noteworthy that, it appears the disproportionately higher risks of mortality from road traffic crashes in months that coincide with festive occasions whereby people who normally reside in cities return to their “hometowns” participate in those festivities which are often characterized by a lot of fanfare and binge drinking. For instance, we notice significantly higher mortality risks during the months of December which coincide with the Christmas season and March/April which seem to coincide with the Easter period.

In conclusion, results reported in this paper suggest the need for immediate and pragmatic steps to be taken to curb the wanton destruction of lives that are occurring on the roads. In particular, there is urgent need to introduce road safety interventions to address this public health hazard that is claiming the lives of young adults.
